# Neratinib + capecitabine sustains health-related quality of life in patients with HER2-positive metastatic breast cancer and ≥ 2 prior HER2-directed regimens

**DOI:** 10.1007/s10549-021-06217-4

**Published:** 2021-04-28

**Authors:** Beverly Moy, Mafalda Oliveira, Cristina Saura, William Gradishar, Sung-Bae Kim, Adam Brufsky, Sara A. Hurvitz, Larisa Ryvo, Daniele Fagnani, Sujith Kalmadi, Paula Silverman, Suzette Delaloge, Jesus Alarcon, Ava Kwong, Keun Seok Lee, Peter Cher Siang Ang, Samuel Guan Wei Ow, Sung-Chao Chu, Richard Bryce, Kiana Keyvanjah, Judith Bebchuk, Bo Zhang, Nina Oestreicher, Ron Bose, Nancy Chan

**Affiliations:** 1 Massachusetts General Hospital Cancer Center, 55 Fruit Street, Boston, MA 02114 USA; 2 Vall D’Hebron University Hospital, Vall D’Hebron Institute of Oncology, Barcelona, Spain; 3 Robert H. Lurie Comprehensive Cancer Center of Northwestern University, Chicago, IL USA; 4 Asan Medical Center, University of Ulsan College of Medicine, Seoul, Korea; 5 Magee-Womens Hospital of UPMC, Pittsburgh, PA USA; 6 University of California Los Angeles/Jonsson Comprehensive Cancer Center, Los Angeles, CA USA; 7 Assuta Ashdod Medical Center, Ashdod, Israel; 8 Azienda Socio-Sanitaria Territoriale Di Vimercate, Vimercate, Italy; 9 Ironwood Cancer and Research Center, Chandler, AZ USA; 10 University Hospitals Cleveland Medical Center, Cleveland, OH USA; 11 Gustave Roussy, Villejuif, France; 12 Servicio de Oncologia, Hospital Universitario Son Espases, Palma de Mallorca, Balearic Islands, Spain; 13 Department of Surgery, Queen Mary Hospital, Hong Kong, Hong Kong; 14 National Cancer Center, Gyeonggi-do, Republic of Korea; 15 Gleneagles Medical Centre, Singapore, Singapore; 16 National University Cancer Institute, Singapore, Singapore; 17 Hualien Tzu Chi Medical Center, Hualien, Taiwan; 18 Puma Biotechnology Inc., Los Angeles, CA USA; 19 Washington University School of Medicine, St. Louis, MO USA; 20 Rutgers Cancer Institute of New Jersey, New Brunswick, NJ USA

**Keywords:** Neratinib, Metastatic breast cancer, Health-related quality of life, HER2-positive

## Abstract

**Purpose:**

To characterize health-related quality of life (HRQoL) in patients with human epidermal growth factor receptor 2 (HER2)-positive metastatic breast cancer (MBC) from the NALA phase 3 study.

**Methods:**

In NALA (NCT01808573), patients were randomized 1:1 to neratinib + capecitabine (N + C) or lapatinib + capecitabine (L + C). HRQoL was assessed using seven prespecified scores from the European Organisation for Research and Treatment of Cancer Quality Of Life Questionnaire core module (QLQ-C30) and breast cancer-specific questionnaire (QLQ-BR23) at baseline and every 6 weeks. Descriptive statistics summarized scores over time, mixed models evaluated differences between treatment arms, and Kaplan–Meier methods were used to assess time to deterioration in HRQoL scores of ≥ 10 points.

**Results:**

Of the 621 patients randomized in NALA, patients were included in the HRQoL analysis if they completed baseline and at least one follow-up questionnaire. The summary, global health status, physical functioning, fatigue, constipation, and systemic therapy side effects scores were stable over time with no persistent differences between treatment groups. There were no differences in time to deterioration (TTD) for the QLQ-C30 summary score between treatment arms; the hazard ratio (HR) for N + C vs. L + C was 0.94 (95% CI 0.63–1.40). Only the diarrhea score worsened significantly more in the N + C arm as compared to the L + C arm, and this remained over time (HR for TTD for N + C vs. L + C was 1.71 [95% CI 1.32–2.23]).

**Conclusion:**

In NALA, patients treated with N + C maintained their global HRQoL over time, despite a worsening of the diarrhea-related scores. These results may help guide optimal treatment selection for HER2-positive MBC.

**Supplementary Information:**

The online version contains supplementary material available at 10.1007/s10549-021-06217-4.

## Introduction

Overall survival in patients with human epidermal growth factor receptor (HER)2-positive metastatic breast cancer (MBC) has continued to improve over the past decade due to advancements in HER2-directed therapies [1, 2], but it remains difficult to treat. Survival is higher for patients with de novo MBC than for those with relapsed disease, and acquired resistance to anti-HER2 therapies remains a challenge [3, 4]. Therefore, the main goals in treating HER2-positive MBC are to prevent tumor progression with minimal toxicity and to preserve patient quality of life (QoL) [5, 6].

Neratinib (Nerlynx^®^; Puma Biotechnology, Inc., Los Angeles, CA) is an irreversible small-molecule tyrosine kinase inhibitor of HER1, HER2, and HER4. Neratinib was first approved as a single agent by the US Food and Drug Administration (FDA) for extended adjuvant treatment of patients with early-stage, HER2-positive breast cancer following adjuvant trastuzumab-based therapy [7], and by the European Medicines Agency for patients with early-stage hormone receptor-positive HER2-overexpressed/amplified breast cancer who completed trastuzumab-based therapy less than one year ago [8]. In 2020, the FDA approved neratinib in combination with capecitabine (N + C) for the treatment of adult patients with advanced or metastatic HER2-positive breast cancer who have received two or more prior anti-HER2 regimens in the metastatic setting [9].

N + C demonstrated efficacy in early clinical studies in metastatic HER2-positive breast cancer [10, 11]. In NALA, N + C significantly prolonged progression-free survival (PFS) compared with lapatinib plus capecitabine (L + C) (hazard ratio [HR] 0.76; translating to a 2.2-month mean PFS improvement) [12]. Safety data in NALA were consistent with previous studies of neratinib in which diarrhea was the most frequently observed treatment-emergent adverse event (grade 3/4 diarrhea in 24% of patients in the N + C arm) [11–13].

Although there are several HER2-directed treatment options for HER2-positive MBC, not all have been well described in terms of their impact on patients’ health-related QoL (HRQoL). The objective of this analysis was to characterize HRQoL among patients with HER2-positive MBC in the NALA clinical study.

## Methods

### Study design and treatment

NALA was a multinational, randomized, open-label, phase 3 clinical trial (Clinicaltrials.gov NCT01808573) comparing N + C against L + C in patients with HER2-positive MBC. The trial design and primary outcomes have been previously described [12]. In brief, eligible patients were aged ≥ 18 years, with an Eastern Cooperative Oncology Group performance status ≤ 1, centrally confirmed HER2-positive MBC [14], and ≥ 2 previous HER2-directed therapies for MBC. Patients were randomized 1:1 to N + C [neratinib 240 mg orally once daily continuously, plus capecitabine 1500 mg/m^2^ orally daily in two evenly spaced doses (750 mg/m^2^ bid) on days 1–14 of 21-day cycles] or to L + C [lapatinib 1250 mg orally once daily continuously, plus capecitabine 2000 mg/m^2^ orally daily in two evenly spaced doses (1000 mg/m^2^ bid) on days 1–14 of 21-day cycles]. The prophylactic antidiarrheal loperamide was required per protocol for all patients in the N + C arm during Cycle 1. In the L + C arm and after Cycle 1 in the N + C group, the use of antidiarrheal prophylaxis was at the discretion of the treating physician. Patients received study treatment until disease progression, unacceptable toxicity, any other specified treatment-withdrawal criterion, or death. The co-primary endpoints of the trial were PFS and overall survival. The protocol was approved by national/institutional ethics committees at participating sites and conducted in accordance with the Declaration of Helsinki. All patients provided written informed consent prior to any protocol-specific procedures or study drug administration.

### HRQoL assessments

Patient-reported HRQoL was a secondary endpoint. HRQoL was measured using the European Organisation for Research and Treatment of Cancer (EORTC) Quality of Life Questionnaire core module (QLQ-C30; version 3), and the EORTC Quality of Life Questionnaire Breast Cancer-Specific Module (QLQ-BR23) [15, 16]. QLQ-C30 and QLQ-BR23 are widely used to measure HRQoL in patients with cancer and include both multi-item scales and single-item measures [17]. The EORTC QLQ-C30 includes a global health status/HRQoL scale, five functional scales (physical, role, emotional, social, and cognitive), and nine symptom scales (fatigue, nausea and vomiting, pain, dyspnea, insomnia, appetite loss, constipation, diarrhea, and financial difficulties). The QLQ-BR23 includes 23 questions addressing four functional areas (body image, sexual functioning, sexual enjoyment, and future perspective) and four symptoms (systemic therapy side effects, upset by hair loss, breast symptoms, and arm symptoms). In NALA, the QLQ-C30 summary score and six scales were prespecified for analysis: global health status (two items), physical functioning (five items), fatigue (three items), constipation (one item), diarrhea (one item), and the QLQ-BR23 systemic therapy side effects scale (seven items). The scales of interest were selected due to the potential impact of study treatment including prophylactic diarrheal treatment. The QLQ-C30 summary scale is calculated from the mean of scores from 13 of the 15 QLQ-C30 scales; the global health status scale and the financial impact scale are not included.

The EORTC assessments were completed electronically by patients before randomization at the baseline visit, then at the beginning of every other 3-week treatment cycle (every 6 weeks ± 3 days) starting from Cycle 3, and finally at treatment discontinuation. Patients indicated the extent to which they had experienced symptoms or problems using a 4-point Likert scale, from 1 = not at all to 4 = very much. The global health status scale used a 7-point Likert scale, on which 1 = very poor and 7 = excellent.

Questionnaire responses are converted to a score ranging from 0 to 100. For the QLQ-C30 summary score and functional scales, higher scores represent better function. A higher score on the global health status scale indicates better HRQoL, whereas for all symptom scores, higher scores indicate a higher level of symptoms. Scoring for the QLQ-BR23 is identical to that for the functional and symptom scales of the QLQ-C30.

### Statistical analyses

For these analyses, a patient was included in the HRQoL analysis population for a particular scale if they had received at least one dose of study drug, had a baseline assessment for that scale, and had at least one post-baseline assessment (up to last dose day + 28 days) for the specific scale.

QLQ-C30 and QLQ-BR23 completion rates were described for each treatment group by visit and defined as the proportion of the expected number of assessments that were actually completed from the baseline visit through to the last post-baseline assessment (last dose date + 28 days).

Changes of ≥ 10 points in HRQoL scores from baseline or between groups were considered clinically meaningful, a change widely regarded as clinically meaningful for the QLQ-C30 in randomized clinical trials [18, 19]. For the functional scales and QLQ-C30 summary score, an improvement is defined as an increase of ≥ 10 points, worsening is defined as a decrease of ≥ 10 points, and stable is defined as neither improved nor worsened. For the symptom scales, an improvement is defined as a decrease of ≥ 10 points, worsening is defined as an increase of ≥ 10 points, and stable is defined as neither improved nor worsened.

Observed scores over time in the prespecified scales were compared descriptively between treatment groups. For the purposes of this report, mean scores were plotted to Cycle 19, which approximates to 1 year of treatment. A time-to-deterioration (TTD) analysis was performed for the seven prespecified scales; this was defined as the time from baseline to the first assessment date with an observed ≥ 10-point decrease (for all functional scales) or increase (for all symptom scales). If the patient’s score change did not reach the deterioration threshold value, they were censored at their last HRQoL assessment. If a patient died (on or before last dose date + 28 days) before a documented decline in HRQoL assessment, the patient was considered to have had the deterioration event on the death date unless the date of death occurred after two missed HRQoL assessments (12 weeks + 3 days); in that case, the patient was censored at the last HRQoL assessment before death. The log-rank test was used to assess treatment differences. In addition, a stratified Cox proportional hazards model was used to estimate the HR. The stratification factors used were prior HER2-directed regimens in the metastatic setting (2 or 3 +), hormone receptor status (positive or negative), and disease location (visceral or nonvisceral only). A mixed-model analysis with an outcome of change from baseline and the covariates, including baseline score, treatment arm, visit (categorical), treatment arm by time interaction, prior HER2-directed regimens in the metastatic setting (2 or 3 +), hormone receptor status (positive or negative), and disease location (visceral or nonvisceral only), was used to evaluate differences between treatment arms over time. The analysis used the F test from the repeated measures mixed model.

All analyses presented are descriptive and no adjustments were made for multiplicity. Analyses were conducted using SAS (version 9.1; SAS Institute, Cary, NC, USA).

## Results

### HRQoL population

Between May 29, 2013, and July 21, 2017, 621 patients were randomized (N + C *n* = 307; L + C *n* = 314). Of the 621 patients, 556 (89.5%; N + C *n* = 275, L + C *n* = 281) had at least one dose of study drug, completed the EORTC QLQ-C30 at baseline and at least once more during follow-up, and formed the QLQ-C30 population. The QLQ-BR23 analysis population comprised a total of 559 patients (90.0%; N + C *n* = 276, L + C *n* = 283). Patient characteristics and demographics of the QLQ-C30 population were well balanced between the two treatment groups (Table 1).

Of the patients who started a treatment cycle, generally over 80% completed each EORTC QLQ-C30 summary score and QLQ-BR23 systemic therapy side effects scale throughout the follow-up and this rate was similar between treatment arms (Online Resource 1). Median treatment duration was 5.7 (interquartile range 2.7–10.4) months for neratinib and 4.4 (interquartile range 2.3–7.1) months for lapatinib.

**Table 1:** Baseline patient demographics and characteristics of the EORTC QLQ-C30 population

Patient baseline characteristic	EORTC QLQ-C30 population (*N* = 556)^a^	
	Neratinib + capecitabine (*n* = 275)	Lapatinib + capecitabine (*n* = 281)
Age, mean (SD)	54 (11.4)	54 (11.4)
Sex, female, *n* (%)	275 (100)	278 (98.9)
Race, *n* (%)		
White	154 (56.0)	154 (54.8)
Asian	105 (38.2)	102 (36.3)
Black	6 (2.2)	9 (3.2)
Other/unknown/missing	9 (3.3)	15 (5.3)
Geographic region, *n* (%)		
Europe	107 (38.9)	112 (39.9)
North America	47 (17.1)	50 (17.8)
Rest of the world	121 (44.0)	119 (42.3)
ECOG performance status at enrollment, *n* (%)		
0	159 (57.8)	149 (53.0)
1	116 (42.2)	132 (47.0)
Hormone receptor status, *n* (%)		
Positive	162 (58.9)	169 (60.1)
Negative	113 (41.1)	112 (39.9)
Number of prior HER2-directed regimens in the metastatic setting, *n* (%)		
2	192 (69.8)	191 (68.0)
≥ 3	83 (30.2)	90 (32.0)
Disease location, *n* (%)		
Visceral	218 (79.3)	227 (80.8)
Nonvisceral	57 (20.7)	54 (19.2)

*EORTC QLQ-C30* European Organisation for Research and Treatment of Cancer Quality of Life Questionnaire core module, *ECOG* Eastern Cooperative Oncology Group, *HER2* human epidermal growth factor receptor 2, *SD* standard deviation

^a^Defined as patients who received at least one dose of study drug, had a baseline assessment for that scale, and had at least one post-baseline assessment (up to last dose day + 28 days) for the specific scale

### EORTC QLQ‑C30 summary score and global health status/QoL scale

**Fig. 1:**
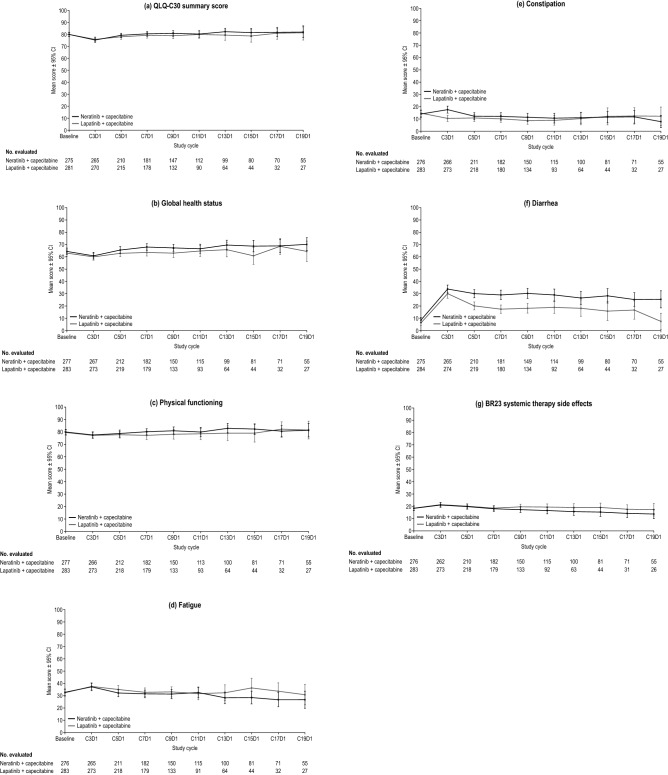
Mean [95% confidence interval (CI)] observed scores over time by treatment group (N + C vs. L + C) in the NALA health-related quality of life population for each prespecified EORTC QLQ-C30 and QLQ-BR23 scale: **a** QLQ-C30 summary score, **b** global health status, **c** physical function, **d** fatigue, **e** constipation, **f** diarrhea, and **g** QLQ-BR23 systemic therapy side effects. For the QLQ-C30 summary score and the physical functioning scale, higher scores represent better function. A higher score on the global health status scale indicates a higher health-related quality of life. For the three symptom scales and the QLQ-BR23 systemic therapy side effects scale, a higher score indicates a higher level of symptoms. *QLQ-BR23* Quality of Life Questionnaire Breast Cancer-Specific Module, *CxDy* Cycle *x* Day *y*, *EORTC* European Organisation for Research and Treatment of Cancer, *L* + *C* lapatinib plus capecitabine, *N* + *C* neratinib plus capecitabine*, QLQ-C30* Quality of Life Questionnaire core module. **a** and **b** are reprinted with permission. Saura et al (2020) J Clin Oncol 38(27): 3138–3149 © 2020 American Society of Clinical Oncology. All rights reserved

At baseline (*n* = 556), the mean [standard deviation (SD)] QLQ-C30 summary scores were similar between the treatment arms [N + C 79.8 (14.1), L + C 79.9 (15.7)] (Table 2). Over time, mean QLQ-C30 summary and global health status/HRQoL scores were similar between treatment arms and remained stable (Fig. 1a, b) [12].

**Table 2:** Baseline EORTC QLQ-C30 and QLQ-BR23 systemic therapy side effects scale scores

QLQ-C30 and QLQ-BR23 scales, (*n* in N + C arm/*n* in L + C arm)	Mean (SD) score	
	N + C	L + C
QLQ-C30 summary score, (275/281)	79.8 (14.1)	79.9 (15.7)
Global health status scale, (277/283)	64.5 (21.5)	63.0 (22.0)
Physical functioning scale, (277/283)	79.9 (18.4)	79.5 (19.5)
Fatigue scale, (276/283)	32.8 (22.5)	32.4 (24.8)
Constipation scale, (276/283)	14.1 (23.9)	14.7 (24.0)
Diarrhea scale, (275/284)	8.6 (16.7)	6.2 (16.0)
QLQ-BR23 systemic therapy side effects scale (276/283)	18.4 (15.9)	18.5 (15.5)

*EORTC* European Organisation for Research and Treatment of Cancer, *L* + *C* lapatinib plus capecitabine, *n* sample size, *N* + *C* neratinib plus capecitabine, *QLQ-BR23* Quality of Life Questionnaire Breast Cancer-Specific Module, *QLQ-C30* Quality of Life Questionnaire core module, *SD* standard deviation

**Fig. 2:**
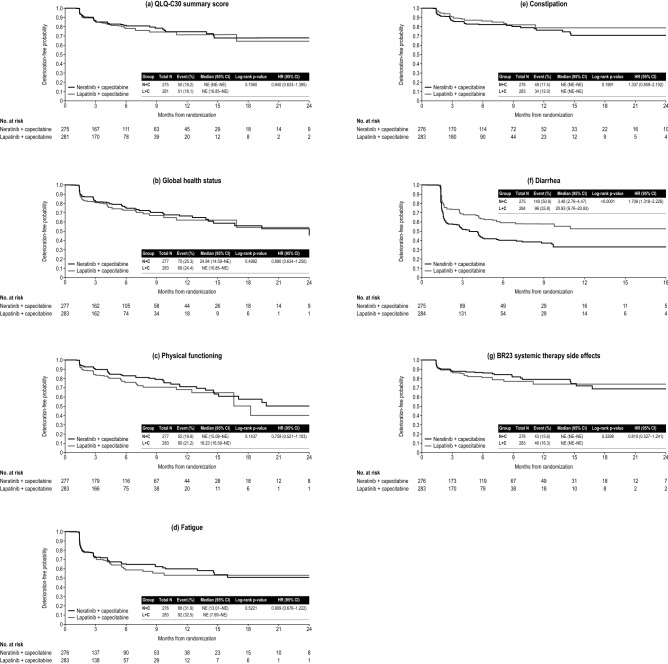
Kaplan–Meier plot for time to deterioration for the first confirmed ≥ 10-point change in **a** EORTC QLQ-C30 summary score and each prespecified scale: **b** global health status, **c** physical functioning, **d** fatigue, **e** constipation, **f** diarrhea, and **g** QLQ-BR23 systemic therapy side effects. *QLQ-BR23* Quality of Life Questionnaire Breast Cancer-Specific Module, *CI* confidence interval*, EORTC* European Organisation for Research and Treatment of Cancer, *HR* hazard ratio, *NE* not estimable*, QLQ-C30* Quality of Life Questionnaire core module

There was no difference in TTD for the QLQ-C30 summary score between treatment arms; the HR for N + C vs. L + C was 0.94 [95% confidence interval (CI) 0.63–1.40] (Fig. 2a). Likewise, the mean global health status scale score remained stable over time and there was no difference between the N + C and L + C arms for TTD on the global health status scale (HR 0.89; 95% CI 0.63–1.25) (Fig. 2b).

### EORTC QLQ-C30 physical functioning scale and symptom scales

For the physical functioning scale, the mean (SD) scores for both treatment arms were maintained from baseline (*n* = 560) over time [N + C 79.9 (18.4), L + C 79.5 (19.5)] (Fig. 1c), indicating that patients did not experience a decrease in their physical function during treatment. Mean (SD) baseline (*n* = 559) scores for the fatigue [N + C 32.8 (22.5), L + C 32.4 (24.8)] and constipation [N + C 14.1 (23.9), L + C 14.7 (24.0)] scales were both at the lower end of each scale’s score range for both treatment arms (Table 2). Scores on the fatigue and constipation symptom scales trended slightly downward from the baseline assessment for both treatment groups over time (Fig. 1d, e).

There was little difference between the two treatment arms in TTD in the physical functioning scale (HR 0.76; 95% CI 0.52–1.10; Fig. 2c) with N + C tending to do better. The TTD in the fatigue or constipation symptom scale did not differ between treatment groups (Fig. 2d, e); HR for TTD with N + C vs. L + C on the fatigue scale was 0.91 (95% CI 0.68–1.22) and 1.34 (95% CI 0.86–2.10) for the constipation scale.

At baseline (*n* = 559), mean (SD) scores on the diarrhea symptom scale were 8.6 (16.7) vs. 6.2 (16.0) in the N + C and L + C arms, respectively (Table 2). From baseline to treatment Cycle 3, mean scores rose on the diarrhea scale—representing an increase in diarrhea symptoms for both treatment arms. Scores rose to a greater extent in the N + C arm but then decreased gradually over time for both arms (Fig. 1f).

The median TTD in the diarrhea symptom scale was shorter for N + C (3.5 months) compared with L + C (20.9 months; HR 1.71; 95% CI 1.32–2.23) (Fig. 2f).

### QLQ-BR23 systemic therapy side effects

Mean (SD) scores on the QLQ-BR23 systemic therapy side effects scale were comparable between treatment arms at baseline (*n* = 559) [N + C 18.4 (15.9), L + C 18.5 (15.5)] (Table 2). Mean scores rose slightly at Cycle 3 in both treatment arms, indicating patients were experiencing an increase in systemic therapy side effects. However, scores decreased gradually for both groups over time (Fig. 1g).

The TTD did not differ between treatment arms (HR 0.81; 95% CI 0.53–1.24) (Fig. 2g).

### Mixed models

Of the seven scales analyzed, only the fatigue scale had a meaningful interaction between visit and treatment group. For the other six scales, a reduced model without the interaction term was analyzed (Online Resource 2). Of these scales, the global health status, constipation, diarrhea, and QLQ-BR23 systemic therapy side effects scales had overall treatment differences, with Global Health Summary, constipation, and diarrhea scales favoring L + C and QLQ-BR23 systemic therapy scale favoring N + C; none met the 10-point difference previously described. The interpretation of the overall treatment effect is the mean difference between the treatment groups over time, and therefore with the diarrhea scale the N + C group had on average a score of 9.3 points greater than the L + C group.

## Discussion

Delaying disease progression for patients while maintaining QoL and minimizing treatment toxicity are key objectives in managing MBC [5]. In the phase 3 NALA study, N + C significantly improved PFS compared with L + C in patients with HER2-positive MBC who had received ≥ 2 prior HER2-directed regimens in the metastatic setting [12]. These patients maintained their HRQoL and functioning throughout the study as measured by the QLQ-C30 summary scale, the global health status scale, and the physical functioning scale, despite the early transient presence of diarrhea symptoms in some patients. Furthermore, symptoms of fatigue and constipation and side effects associated with systemic therapy remained stable throughout treatment.

Although mean overall QoL (QLQ-C30 summary and global health status scores) declined slightly from baseline to the next assessment at Cycle 3, by Cycle 5 patients reported similar levels of overall functioning and symptoms compared to those at the baseline visit. On average, this return to their overall baseline level of functioning and symptoms remained stable for patients throughout follow-up. The slight initial change at the beginning of treatment and then return to pretreatment levels may indicate that patients felt an increase in symptoms and an impact on function at the start of treatment, but that this effect was transient and followed the clinical course of diarrhea. This initial short-term impairment in HRQoL at the start of therapy followed by recovery to baseline levels has been observed previously in the metastatic setting [20]. The phase 3 CLEOPATRA trial (NCT00567190) studied the addition of pertuzumab to trastuzumab plus docetaxel in patients with previously untreated HER2-positive MBC. CLEOPATRA included a composite trial outcome index, comprising physical well-being, functional well-being, and breast cancer-specific scales; mean scores appeared to worsen in both treatment arms from baseline to Week 18 (treatment Cycle 6), after which scores recovered to baseline levels and in the pertuzumab arm appeared to improve after Week 63 [20].

As there are other treatment options beyond the second line for patients with HER2-positive MBC, it is important to have comprehensive QoL data to help inform patient treatment decisions. In addition to approving neratinib in third-line MBC, the FDA recently approved trastuzumab deruxtecan (DS-8201; Daiichi Sankyo and AstraZeneca) and tucatinib (Seattle Genetics) [21, 22]. Although HRQoL was not measured in the pivotal trastuzumab deruxtecan trial, DESTINY-Breast01, other trials in the program have included the QLQ-C30 and QLQ-BR45 (the updated version of QLQ-BR23) [23]. The HER2Climb study (NCT02614794) of tucatinib vs. trastuzumab and capecitabine for HER2-positive MBC included the EuroQol 5-Dimensions 5-Levels (EQ-5D-5L) questionnaire, a brief generic health instrument, to measure HRQoL [24].

A strength of the NALA study is its comprehensive evaluation of the impact of treatment on HRQoL, a key clinical outcome for patients with MBC in the third line of treatment and beyond. This evaluation was conducted with validated instruments commonly used in breast cancer and is strengthened by the prespecified complementary set of analyses including mean scores over time and mixed-model analyses examining differences in treatment groups over time. The study is further enhanced by the inclusion of TTD analysis using a threshold of ≥ 10 points, a change widely regarded as clinically meaningful for the QLQ-C30 in randomized clinical trials [19]. The TTD analysis provides a view of changes in individual patients instead of simply evaluating mean change over time by treatment arm.

A limitation of these data is that, following the baseline assessment, the EORTC QLQ-C30 was not collected again until Cycle 3; therefore, the instrument may not have captured the full pattern of HRQoL during the first and second treatment cycles, and particularly the pattern of an early transient presence of diarrhea in some patients. Although the overall adverse-event profile was similar between regimens and diarrhea was the most prevalent adverse event in both treatment arms, diarrhea occurred more frequently in the N + C arm, particularly during the first treatment cycle. At Cycle 3, responses to the EORTC QLQ-C30 diarrhea scale reflected the occurrence of more diarrhea being reported in the N + C group compared with the L + C arm. However, treatment discontinuation rates due to diarrhea were low and approximately equivalent between treatment groups; rates of discontinuation due to any treatment-emergent adverse event were lower in N + C-treated patients than in patients treated with L + C [12]. Furthermore, with combination therapy it is not possible to determine which component of the treatment regimen has the greatest influence on the aspects of function and symptoms as measured by the EORTC QLQ-C30 and QLQ-BR23.

In conclusion, these results from the NALA trial in patients with HER2-positive MBC demonstrate that treatment with N + C sustains patient HRQoL while improving PFS over L + C, despite an early impact on patient-reported diarrhea. These results may help guide healthcare providers and patients in the selection of optimal treatments for HER2 + MBC.

## Supplementary Information

Below is the link to the electronic supplementary material.

Supplementary file 1 (DOCX 21 kb)

## Data Availability

The authors declare that the data supporting the findings of this study are available within the article. The authors may be contacted for further data sharing.
