# Supplementary material for: Neratinib + capecitabine sustains health-related quality of life in patients with HER2-positive metastatic breast cancer and ≥ 2 prior HER2-directed regimens
Source: Breast Cancer Res Treat. 2021 Apr 28;188(2):449–58. doi: 10.1007/s10549-021-06217-4 (PMC8260518; doi:10.1007/s10549-021-06217-4)
Supplement: Supplementary file 1 — (DOCX 21 kb) [file 10549_2021_6217_MOESM1_ESM.docx]

## Neratinib + capecitabine sustains health-related quality of life while improving progression-free survival in patients with HER2-positive metastatic breast cancer and ≥2 prior HER2-directed regimens

Breast Cancer Research and Treatment

Beverly Moy^1^ · Mafalda Oliveira^2^ · Cristina Saura^2^ · William Gradishar^3^ · Sung-Bae Kim^4^ · Adam Brufsky^5^ · Sara A. Hurvitz^6^ · Larisa Ryvo^7^ · Daniele Fagnani^8^ · Sujith Kalmadi^9^ · Paula Silverman^10^ · Suzette Delaloge^11^ · Jesus Alarcon^12^ · Ava Kwong^13^ · Keun Seok Lee^14^ · Peter Cher Siang Ang^15^ · Samuel Guan Wei Ow^16^ · Sung-Chao Chu^17^ · Richard Bryce^18^ · Kiana Keyvanjah^18^ · Judith Bebchuk^18^ · Bo Zhang^18^ · Nina Oestreicher^18^ · Ron Bose^19^ · Nancy Chan^20^

^1^ Massachusetts General Hospital Cancer Center, Boston, MA, USA

^2^ Vall d’Hebron University Hospital, Vall d’Hebron Institute of Oncology, Barcelona, Spain

^3^ Robert H. Lurie Comprehensive Cancer Center of Northwestern University, Chicago, IL, USA

^4^ Asan Medical Center, University of Ulsan College of Medicine, Seoul, Korea

^5^ Magee-Womens Hospital of UPMC, Pittsburgh, PA, USA

^6^ University of California Los Angeles/Jonsson Comprehensive Cancer Center, Los Angeles, CA, USA

^7^ Assuta Ashdod Medical Center, Ashdod, Israel

^8^ Azienda Socio-Sanitaria Territoriale di Vimercate, Vimercate, Italy

^9^ Ironwood Cancer and Research Center, Chandler, AZ, USA

^10^ University Hospitals Cleveland Medical Center, Cleveland, OH, USA

^11^ Gustave Roussy, Villejuif, France

^12^ Hospital Universitario Son Espases, Servicio de Oncologia, Palma de Mallorca, Balearic Islands, Spain

^13^ Queen Mary Hospital, Department of Surgery, Hong Kong

^14^ National Cancer Center, Gyeonggi-do, Republic of Korea

^15^ Gleneagles Medical Centre, Singapore

^16^ National University Cancer Institute, Singapore

^17^ Hualien Tzu Chi Medical Center, Hualien, Taiwan

^18^ Puma Biotechnology Inc., Los Angeles, CA, USA

^19^ Washington University School of Medicine, St. Louis, MO, USA

^20^ Rutgers Cancer Institute of New Jersey, New Brunswick, NJ, USA

🖂 Beverly Moy
[bmoy@mgh.harvard.edu](mailto:bmoy@mgh.harvard.edu)

Massachusetts General Hospital Cancer Center, 55 Fruit Street, 02114, Boston, MA, USA; Telephone: +1 617 726 5130

**Online Resource 1** Proportion of patients completing the EORTC QLQ-C30 summary score and QLQ-BR23 systemic therapy side effects scale at scheduled assessments

| EORTC assessment | Patients completing, % (*n*/*N*) | | |  |
| --- | --- | --- | --- | --- |
|  | N+C | L+C |  |  |
| **QLQ-C30 summary score** |  |  | |  |
| Baseline | 100.0 (275/275) | 100.0 (281/281) | |  |
| Cycle 3 | 96.4 (265/275) | 96.1 (270/281) | |  |
| Cycle 5 | 84.0 (210/250) | 84.6 (215/254) | |  |
| Cycle 7 | 87.9 (181/206) | 87.3 (178/204) | |  |
| Cycle 9 | 83.1 (147/177) | 81.0 (132/163) | |  |
| Cycle 11 | 75.7 (112/148) | 75.6 (90/119) | |  |
| Cycle 13 | 83.2 (99/119) | 76.2 (64/84) | |  |
| Cycle 15 | 83.3 (80/96) | 71.0 (44/62) | |  |
| Cycle 17 | 88.6 (70/79) | 78.0 (32/41) | |  |
| Cycle 19 | 83.3 (55/66) | 84.4 (27/32) | |  |
| **QLQ-BR23 systemic therapy side effects** | | | | |
| Baseline | 100.0 (276/276) | 100.0 (283/283) | |  |
| Cycle 3 | 94.9 (262/276) | 96.5 (273/283) | |  |
| Cycle 5 | 83.7 (210/251) | 85.2 (218/256) | |  |
| Cycle 7 | 87.9 (182/207) | 87.3 (179/205) | |  |
| Cycle 9 | 84.3 (150/178) | 81.1 (133/164) | |  |
| Cycle 11 | 77.2 (115/149) | 76.7 (92/120) | |  |
| Cycle 13 | 83.3 (100/120) | 75.0 (63/84) | |  |
| Cycle 15 | 83.5 (81/97) | 72.1 (44/61) | |  |
| Cycle 17 | 88.8 (71/80) | 77.5 (31/40) | |  |
| Cycle 19 | 82.1 (55/67) | 83.9 (26/31) | |  |

*EORTC* European Organization for Research and Treatment of Cancer, *L+C* lapatinib plus capecitabine, *N+C* neratinib plus capecitabine, *QLQ-BR23* Quality of Life Questionnaire Breast Cancer-Specific Module, *QLQ-C30* Quality of Life Questionnaire core module

Neratinib + capecitabine sustains health-related quality of life while improving progression-free survival in patients with HER2-positive metastatic breast cancer and ≥2 prior HER2-directed regimens

Breast Cancer Research and Treatment

Beverly Moy^1^ · Mafalda Oliveira^2^ · Cristina Saura^2^ · William Gradishar^3^ · Sung-Bae Kim^4^ · Adam Brufsky^5^ · Sara A. Hurvitz^6^ · Larisa Ryvo^7^ · Daniele Fagnani^8^ · Sujith Kalmadi^9^ · Paula Silverman^10^ · Suzette Delaloge^11^ · Jesus Alarcon^12^ · Ava Kwong^13^ · Keun Seok Lee^14^ · Peter Cher Siang Ang^15^ · Samuel Guan Wei Ow^16^ · Sung-Chao Chu^17^ · Richard Bryce^18^ · Kiana Keyvanjah^18^ · Judith Bebchuk^18^ · Bo Zhang^18^ · Nina Oestreicher^18^ · Ron Bose^19^ · Nancy Chan^20^

^1^ Massachusetts General Hospital Cancer Center, Boston, MA, USA

^2^ Vall d’Hebron University Hospital, Vall d’Hebron Institute of Oncology, Barcelona, Spain

^3^ Robert H. Lurie Comprehensive Cancer Center of Northwestern University, Chicago, IL, USA

^4^ Asan Medical Center, University of Ulsan College of Medicine, Seoul, Korea

^5^ Magee-Womens Hospital of UPMC, Pittsburgh, PA, USA

^6^ University of California Los Angeles/Jonsson Comprehensive Cancer Center, Los Angeles, CA, USA

^7^ Assuta Ashdod Medical Center, Ashdod, Israel

^8^ Azienda Socio-Sanitaria Territoriale di Vimercate, Vimercate, Italy

^9^ Ironwood Cancer and Research Center, Chandler, AZ, USA

^10^ University Hospitals Cleveland Medical Center, Cleveland, OH, USA

^11^ Gustave Roussy, Villejuif, France

^12^ Hospital Universitario Son Espases, Servicio de Oncologia, Palma de Mallorca, Balearic Islands, Spain

^13^ Queen Mary Hospital, Department of Surgery, Hong Kong

^14^ National Cancer Center, Gyeonggi-do, Republic of Korea

^15^ Gleneagles Medical Centre, Singapore

^16^ National University Cancer Institute, Singapore

^17^ Hualien Tzu Chi Medical Center, Hualien, Taiwan

^18^ Puma Biotechnology Inc., Los Angeles, CA, USA

^19^ Washington University School of Medicine, St. Louis, MO, USA

^20^ Rutgers Cancer Institute of New Jersey, New Brunswick, NJ, USA

🖂 Beverly Moy
[bmoy@mgh.harvard.edu](mailto:bmoy@mgh.harvard.edu)

Massachusetts General Hospital Cancer Center, 55 Fruit Street, 02114, Boston, MA, USA; Telephone: +1 617 726 5130

**Online Resource 2** Mixed-model analysis results (change from baseline)

|  | Overall Treatment Difference Estimate | p-value^a^ |
| --- | --- | --- |
| QLQ-C30 Summary Score^b^ |  |  |
| Treatment (N+C vs. L+C) | 0.59 | 0.5147 |
| Visit^c^ |  | 0.0067 |
| Global health status scale |  |  |
| Treatment (N+C vs. L+C) | 2.20 | 0.0923 |
| Visit^c^ |  | 0.0360 |
| Physical functioning scale |  |  |
| Treatment (N+C vs. L+C) | 0.77 | 0.5219 |
| Visit^c^ |  | 0.1644 |
| Fatigue scale |  |  |
| Treatment (N+C vs. L+C)^b^ |  | 0.0144 |
| Visit^c^ |  | 0.0893 |
| Treatment Interaction with Visit^c^ |  | 0.0111 |
| Constipation scale |  |  |
| Treatment (N+C vs. L+C) | 2.89 | 0.0291 |
| Visit^c^ |  | 0.2208 |
| Diarrhea scale |  |  |
| Treatment (N+C vs. L+C) | 9.30 | <0.0001 |
| Visit^c^ |  | <0.0001 |
| BR23 systemic therapy side effects scale |  |  |
| Treatment (N+C vs. L+C) | -0.77 | 0.3805 |
| Visit^c^ |  | 0.0021 |

^a^ p-value is from type 3 test of fixed effect.

^b^ Treatment effect is not meaningful in the presence of a significant interaction and is not shown.

^c^ Visit or visit by treatment interaction effect is not the effect estimate of interest and is not shown.

*QLQ-BR23* Quality of Life Questionnaire Breast Cancer-Specific Module, *EORTC* European Organisation for Research and Treatment of Cancer, *L+C* lapatinib plus capecitabine, *N+C* neratinib plus capecitabine, *QLQ-C30* Quality of Life Questionnaire core module
